# Supplementary material for: Potential for risk reduction of chronic health conditions through lifestyle in childhood cancer survivors
Source: Nat Commun. 2026 May 29;17:4605. doi: 10.1038/s41467-026-73517-y (PMC13221451; doi:10.1038/s41467-026-73517-y)
Supplement: Supplementary file 1 — Supplementary Information [file 41467_2026_73517_MOESM1_ESM.pdf]

Supplementary table 1. Number of events by lifestyle score category.

**Outcome**	**Unhealthy lifestyle**		**Moderately healthy lifestyle**		**Healthy lifestyle**	
	N (events)	N (%) pre-existing conditions excluded	N (events)	N (%) pre-existing conditions excluded	N (events)	N (%) pre-existing conditions excluded
**Chronic health conditions**						
Hypertension	6283 (909)	379 (7.9)	7409 (1037)	488 (6.1)	3889 (490)	216 (3.6)
Dyslipidemia	6641 (721)	163 (3.4)	7543 (898)	264 (3.3)	3941 (460)	112 (1.9)
Diabetes mellitus	6822 (386)	142 (3.0)	7599 (361)	193 (2.4)	3815 (116)	93 (1.6)
Heart attack	7008 (244)	50 (1.0)	7649 (249)	85 (1.1)	3843 (108)	29 (0.5)
Heart failure	6993 (206)	68 (1.4)	7611 (246)	103 (1.3)	3832 (118)	57 (1.0)
Stiff or leaky valves	7079 (98)	5 (0.1)	7708 (133)	16 (0.2)	3849 (47)	7 (0.1)
Arrhythmia	6973 (198)	66 (1.4)	7605 (240)	119 (1.5)	3823 (116)	78 (1.3)
Stroke	6899 (186)	108 (2.3)	7481 (248)	232 (2.9)	3814 (104)	130 (2.2)
Joint replacement	6952 (90)	123 (2.6)	7521 (83)	194 (2.4)	3766 (34)	108 (1.8)
Osteoporosis	6894 (188)	108 (2.3)	7481 (257)	237 (3.0)	3812 (106)	132 (2.2)
Respiratory disease	6750 (152)	240 (5.0)	7398 (176)	329 (4.1)	3699 (92)	248 (4.2)
Subsequent malignant neoplasms	6847 (696)	90 (1.9)	7523 (907)	210 (2.6)	3861 (478)	133 (2.2)
**Emotional distress and quality of life**						
Anxiety	4813 (360)	431 (8.2)	6108 (317)	344 (5.3)	3444 (140)	147 (4.1)
Depression	4552 (455)	692 (13.2)	5919 (443)	536 (8.3)	3397 (201)	194 (5.4)
Physical component summary scale	4990 (1173)	61 (1.2)	6389 (1076)	54 (0.8)	3638 (321)	13 (0.4)
Mental component summary scale	4909 (1203)	142 (2.8)	6314 (1104)	129 (2.0)	3606 (504)	45 (1.2)

Supplementary Table 2. Associations between the lifestyle score and chronic health conditions by attained age.

			<30		30-39		40-49		50+	
**Outcome**	**P Interaction**		**Unhealthy**	**Moderate**	**Unhealthy**	**Moderate**	**Unhealthy**	**Moderate**	**Unhealthy**	**Moderate**
Hypertension	0.038	RR	1.3	1.2	1.8	1.5	1.4	1.3	0.9	0.9
		95%CI	1.0-1.7	0.9-1.5	1.5-2.2	1.3-1.9	1.2-1.8	1.0-1.5	0.6-1.2	0.7-1.3
Dyslipidemia	0.40	RR	1.3	1.2	1.3	1.4	1.2	1.1	0.9	1.0
		95%CI	0.9-1.8	0.9-1.5	1.1-1.6	1.2-1.7	1.0-1.5	0.9-1.3	0.6-1.3	0.7-1.4
Diabetes mellitus	0.83	RR	2.4	1.8	2.0	1.7	3.0	2.2	3.3	2.3
		95%CI	1.5-4.0	1.1-2.9	1.5-2.8	1.3-2.3	1.9-4.6	1.4-3.4	1.4-7.6	1.0-5.5
Heart attack	0.17	RR	0.7	0.7	2.1	1.4	1.2	1.2	1.3	1.6
		95%CI	0.3-2.0	0.3-1.5	1.3-3.3	0.9-2.3	0.8-1.7	0.8-1.6	0.8-2.2	1.0-2.8
Heart failure	0.58	RR	1.7	1.2	1.1	1.0	1.2	1.2	1.5	1.9
		95%CI	0.9-3.2	0.7-2.1	0.7-1.6	0.7-1.5	0.8-1.7	0.9-1.8	0.8-3.0	1.0-3.7
Stiff or leaky valves	0.11	RR	6.2	7.8	1.7	1.0	1.7	2.0	0.8	1.1
		95%CI	0.5-78.0	0.9-66.9	0.7-4.0	0.4-2.5	0.9-3.0	1.2-3.5	0.4-1.3	0.7-1.9
Arrhythmia	0.98	RR	1.0	1.1	1.1	1.3	1.3	1.3	1.1	1.2
		95%CI	0.5-2.0	0.6-1.8	0.7-1.7	0.9-2.0	0.9-1.9	0.8-1.9	0.6-1.9	0.7-2.1
Stroke	0.23	RR	0.8	0.9	0.9	1.2	1.6	2.0	1.0	0.9
		95%CI	0.4-1.4	0.5-1.5	0.6-1.4	0.8-1.8	0.9-2.6	1.2-3.4	0.5-2.0	0.4-1.9
Joint replacement	0.39	RR	1.3	0.7	1.6	2.3	1.6	1.2	2.4	1.6
		95%CI	0.5-3.6	0.2-1.9	0.7-3.6	1.1-4.9	0.8-3.1	0.6-2.3	0.9-6.3	0.6-4.4
Osteoporosis	0.14	RR	0.8	0.8	0.9	1.3	1.6	2.1	0.9	0.9
		95%CI	0.4-1.4	0.5-1.4	0.6-1.4	0.9-1.9	1.0-2.7	1.3-3.5	0.4-1.8	0.4-1.8
Respiratory disease	0.59	RR	1.6	1.0	1.0	1.1	1.0	1.1	1.5	1.2
		95%CI	1.0-2.8	0.6-1.6	0.6-1.6	0.7-1.7	0.6-1.8	0.6-1.8	0.7-3.3	0.6-2.7
Subsequent malignant neoplasms	0.008	RR	1.2	1.4	0.8	1.1	0.8	0.9	1.2	1.0
		95%CI	0.8-1.9	1.0-1.9	0.7-1.0	0.9-1.4	0.7-1.0	0.7-1.1	0.9-1.7	0.7-1.4

Models were adjusted for attained age as cubic splines, sex, race/ethnicity, age at cancer diagnosis, socioeconomic status (SES) including education, marriage status, household income, and insurance, with treatment variables chosen from backward selection. Number of participants in the total population is 18,664, with analysis of each outcome differing slightly due to pre-existing conditions, as reported in Supplementary Table 1.
CI=confidence interval. RR=rate ratio.

Supplementary Table 3. Associations between the lifestyle score and emotional distress or impaired quality of life by attained age.

			<30		30-39		40-49		50+	
**Outcome**	**P Interaction**		**Unhealthy**	**Moderate**	**Unhealthy**	**Moderate**	**Unhealthy**	**Moderate**	**Unhealthy**	**Moderate**
Anxiety	0.69	OR	1.6	1.4	1.9	1.4	1.9	1.4	2.0	1.6
		95%CI	1.1-2.4	1.0-1.9	1.5-2.5	1.1-1.8	1.4-2.6	1.0-1.9	1.0-3.9	0.8-3.1
Depression	0.23	OR	1.4	1.3	1.7	1.3	2.0	1.5	1.1	0.9
		95%CI	1.0-2.0	1.0-1.7	1.4-2.2	1.1-1.6	1.6-2.7	1.2-2.0	0.7-1.7	0.6-1.5
Physical quality of life	0.33	OR	1.7	1.2	2.0	1.7	2.3	1.7	2.3	1.8
		95%CI	1.2-2.4	0.9-1.6	1.6-2.4	1.4-2.0	1.9-2.8	1.4-2.0	1.7-3.0	1.4-2.4
Mental quality of life	0.35	OR	1.6	1.2	2.0	1.3	1.9	1.3	1.4	1.1
		95%CI	1.2-2.1	0.9-1.4	1.7-2.4	1.2-1.6	1.5-2.2	1.1-1.6	1.0-1.8	0.8-1.5

Models were adjusted for attained age as cubic splines, sex, race/ethnicity, age at cancer diagnosis, socioeconomic status (SES) including education, marriage status, household income, and insurance, with treatment variables chosen from backward selection. Number of participants in the total population is 18,664, with analysis of each outcome differing slightly due to pre-existing conditions, as reported in Supplementary Table 1.
CI=confidence interval. OR=odds ratio.

Supplementary table 4. Associations between each of the four lifestyle factors and the chronic health conditions.

	Drinking		Smoking		BMI (ref=Normal)						MET (Reference 9+)			
					Underweight		Overweight		Obesity		0-2		3-8	
**Chronic Health Conditions**	**RR**	**95% CI**	**RR**	**95% CI**	**RR**	**95% CI**	**RR**	**95% CI**	**RR**	**95% CI**	**RR**	**95% CI**	**RR**	**95% CI**
Hypertension	1.0	0.9 - 1.1	0.9	0.8 - 1.0	0.6	0.4 - 0.8	1.4	1.3 - 1.6	2.4	2.1 - 2.7	1.1	1.0 - 1.2	1.1	0.9 - 1.2
Dyslipidemia	0.9	0.7 - 1.0	0.9	0.8 - 1.0	0.8	0.5 - 1.1	1.3	1.2 - 1.5	1.7	1.5 - 1.9	1.2	1.1 - 1.4	1.2	1.1 - 1.4
Diabetes mellitus	1.0	0.8 - 1.3	0.8	0.7 - 1.0	0.8	0.5 - 1.5	2.6	2.0 - 3.2	6.3	5.0 - 7.7	1.3	1.0 - 1.5	1.2	1.0 - 1.5
Heart attack	0.9	0.7 - 1.1	1.1	0.9 - 1.4	1.2	0.7 - 1.8	1.2	0.9 - 1.4	1.6	1.2 - 2.0	1.1	0.9 - 1.3	1.0	0.8 - 1.3
Heart failure	1.2	0.9 - 1.6	1.0	0.8 - 1.3	1.3	0.9 - 2.0	1.1	0.9 - 1.5	1.2	0.9 - 1.5	1.4	1.1 - 1.7	1.0	0.7 - 1.3
Stiff or leaky valves	1.1	0.7 - 1.6	1.1	0.9 - 1.5	0.8	0.4 - 1.8	1.6	1.2 - 2.2	2.0	1.4 - 2.8	1.2	0.9 - 1.6	0.9	0.6 - 1.3
Arrhythmia	1.0	0.7 - 1.3	1.0	0.8 - 1.2	1.0	0.6 - 1.6	1.0	0.8 - 1.3	1.5	1.1 - 1.9	1.0	0.8 - 1.3	0.9	0.7 - 1.2
Stroke	0.6	0.4 - 0.8	1.3	1.0 - 1.6	1.1	0.6 - 2.0	1.1	0.8 - 1.3	1.1	0.9 - 1.5	1.0	0.8 - 1.3	1.1	0.8 - 1.5
Joint replacement	1.4	1.0 - 2.0	1.0	0.7 - 1.4	1.3	0.6 - 2.8	1.5	1.1 - 2.2	1.9	1.3 - 2.8	1.3	0.9 - 1.8	1.4	0.9 - 2.1
Osteoporosis	0.6	0.5 - 0.9	1.3	1.0 - 1.6	1.1	0.7 - 2.0	1.0	0.8 - 1.3	1.0	0.8 - 1.4	1.0	0.8 - 1.3	1.1	0.9 - 1.5
Respiratory disease	1.0	0.7 - 1.3	1.0	0.8 - 1.3	0.7	0.4 - 1.3	1.2	0.9 - 1.5	1.3	1.0 - 1.7	1.3	1.0 - 1.7	1.2	0.9 - 1.6
Subsequent malignant neoplasms	0.9	0.8 - 1.0	0.9	0.8 - 1.0	0.9	0.7 - 1.2	1.1	1.0 - 1.3	1.1	0.9 - 1.2	1.1	1.0 - 1.2	1.2	1.0 - 1.3

Models included all four lifestyle variables and were adjusted for attained age as cubic splines, sex, race/ethnicity, age at cancer diagnosis, socioeconomic status (SES) including education, marriage status, household income, and insurance, with treatment variables chosen from backward selection. Number of participants in the total population is 18,664, with analysis of each outcome differing slightly due to pre-existing conditions, as reported in Supplementary Table 1.

BMI=body mass index. CI=confidence interval. RR=rate ratio. MET=metabolic equivalence of task, hours per week.

Supplementary table 5. Associations between each of the four lifestyle factors and the chronic health conditions.

	Drinking		Smoking		BMI (ref=Normal)						MET (Reference 9+)			
					Underweight		Overweight		Obesity		0-2		3-8	
**Emotional distress and quality of life**	**OR**	**95% CI**	**OR**	**95% CI**	**OR**	**95% CI**	**OR**	**95% CI**	**OR**	**95% CI**	**OR**	**95% CI**	**OR**	**95% CI**
Anxiety	1.3	1.1 - 1.6	1.7	1.4 – 1.9	1.1	0.8 - 1.6	1.0	0.9 - 1.2	1.2	1.0 - 1.4	1.0	0.8 - 1.2	0.9	0.8 - 1.1
Depression	1.0	0.9 - 1.3	1.4	1.2 - 1.6	1.0	0.8 - 1.4	1.0	0.9 - 1.1	1.2	1.0 - 1.4	1.2	1.1 - 1.4	1.1	0.9 - 1.3
Physical quality of life	0.9	0.8 - 1.0	1.2	1.1 - 1.4	1.4	1.0 - 1.8	1.1	1.0 - 1.2	1.6	1.4 - 1.8	1.7	1.5 - 1.9	1.1	1.0 - 1.3
Mental quality of life	1.1	1.0 - 1.2	1.5	1.4 - 1.6	1.1	0.9 - 1.4	1.1	1.0 - 1.2	1.2	1.1 - 1.4	1.3	1.2 - 1.5	1.1	1.0 - 1.3

Models included all four lifestyle variables and were adjusted for attained age as cubic splines, sex, race/ethnicity, age at cancer diagnosis, socioeconomic status (SES) including education, marriage status, household income, and insurance, with treatment variables chosen from backward selection. Number of participants in the total population is 18,664, with analysis of each outcome differing slightly due to pre-existing conditions, as reported in Supplementary Table 1.

BMI=body mass index. CI=confidence interval. OR=odds ratio. MET=metabolic equivalence of task, hours per week.

Supplementary table 6. Population attributable fractions for lifestyle, radiation, and chemotherapy, and the treatment exposures included for each outcome.

Outcome	Lifestyle	RT	Chemo	Treatment exposures in final model, resulted from backward selection
	PAF, %	PAF, %	PAF, %	
**Chronic Health Conditions**				
Hypertension	22.1	9.5	9.7	Alkylating agents, chest RT, abdominal RT
Dyslipidemia	14.8	23.3	7.7	Alkylating agents, bleomycine, brain RT, abdominal RT
Diabetes mellitus	51.3	27.3	19.8	Alkylating agents, bleomycine, epipodophyllotoxins, abdominal RT, CNS tumor
Heart attack	15.6	61.4	0.0	Heart RT
Heart failure	19.5	41.5	42.1	Anthracyclines, heart RT
Stiff or leaky valves	35.2	71.7	0.0	Heart RT
Arrhythmia	NA	39.3	18.3	Alkylating agents, anthracyclines, heart RT
Stroke	NA	52.4	6.7	Bleomycine, epipodophyllotoxins, brain RT, chest RT, CNS tumor
Joint replacement	28.3	6.1	2.6	Ifosfamide, leg RT, bone sarcoma
Osteoporosis	NA	46.0	0.0	Hematopoietic stem cell transplantation, brain RT, CNS tumor
Respiratory disease	NA	22.8	0.0	Chest RT
Subsequent malignant neoplasms	NA	38.5	6.9	Anthracyclines, epipodophyllotoxins, abdominal RT, brain RT, chest RT, neck RT
**Emotional distress and quality of life**				
Anxiety	26.1	NA	5.1	Methotrexate intrathecally, brain RT
Depression	22.2	NA	NA	Brain RT
Physical quality of life	30.2	11.1	4.6	Alkylating agent, anthracycline, platinum derivates, epipodophyllotoxins, brain RT, chest RT, pelvis RT
Mental quality of life	21.5	NA	2.5	Methotrexate intrathecally brain RT

Models included lifestyle score, radiotherapy, and chemotherapy exposures and were adjusted for attained age as cubic splines, sex, race/ethnicity, age at cancer diagnosis, socioeconomic status (SES) including education, marriage status, household income, and insurance. Number of participants in the total population is 18,664, with analysis of each outcome differing slightly due to pre-existing conditions, as reported in Supplementary Table 1.
NA=Not applicable, due to no positive association. PAF=population attributable fraction. RT=radiotherapy.

Supplementary table 7. Population attributable fractions for the outcomes for each lifestyle variable.

	**Drinking**	**Smoking**	**BMI**	**MET**
**Chronic Health Conditions**	PAF	PAF	PAF	PAF
Hypertension	NA	NA	26.4	5.0
Dyslipidemia	NA	NA	18.9	12.0
Diabetes mellitus	NA	NA	58.8	13.7
Heart attack	NA	NA	14.2	NA
Heart failure	NA	NA	NA	13.8
Stiff or leaky valves	NA	NA	25.2	NA
Arrhythmia	NA	NA	8.9	NA
Stroke	NA	6.9	NA	NA
Joint replacement	6.4	NA	26.4	NA
Osteoporosis	NA	7.1	NA	NA
Respiratory disease	NA	NA	8.1	15.6
Subsequent malignant neoplasms	NA	NA	NA	7.4
**Emotional distress and quality of life**				
Anxiety	5.5	16.1	4.8	NA
Depression	NA	9.9	3.3	8.5
Physical quality of life	NA	4.9	11.9	20.3
Mental quality of life	1.3	9.8	4.8	11.3

Models included all four lifestyle variables and were adjusted for attained age as cubic splines, sex, race/ethnicity, age at cancer diagnosis, socioeconomic status (SES) including education, marriage status, household income, and insurance, with treatment variables chosen from backward selection. Number of participants in the total population is 18,664, with analysis of each outcome differing slightly due to pre-existing conditions, as reported in Supplementary Table 1.

BMI=body mass index. MET=metabolic equivalence of task, hours per week. NA=Not applicable, due to no positive association. PAF=population attributable fraction.

Supplementary Table 8. Demographic and baseline characteristics in childhood cancer survivors and sibling controls.

	**Survivors N=18664**	**Siblings N=3951**
Sex, Female	9114 (48.9)	2136 (54.1)
Age At Start of Follow-Up, Median (IQR)	25.2 (21.9–30.0)	28.0 (22.9-34.2)
Years Of Follow-Up from Start of follow-up, Median (IQR)	12.9 (9.9-24.9)	15.3 (6.7-22.0)
Race/Ethnicity		
Non-Hispanic White	15297 (81.2)	3475 (88.0)
Non-Hispanic Black	954 (5.3)	102 (2.6)
Hispanic	1332 (7.9)	150 (3.8)
Other	1081 (5.7)	224 (5.7)
Educational Attainment		
≤High school graduate or GED	5417 (29.5)	835 (21.3)
Some college	6988 (38.0)	1462 (37.2)
College graduate or more	6141 (32.5)	1631 (41.5)
Household Income, USD per year		
<20,000	4003 (25.2)	404 (10.4)
20-39,000	2564 (14.5)	362 (9.3)
40-79,000	4609 (24.8)	1012 (26.1)
≥80,000	6840 (35.5)	2098 (54.1)
Marital Status		
Married/living as married	6709 (34.8)	2029 (51.5)
Separated/divorced/Widowed	1117 (5.9)	282 (7.2)
Never married/lived as married	10777 (59.3)	1628 (41.3)
Has health Insurance	15916 (85.0)	3550 (89.9)
Smoking History		
Never	13204 (71.9)	2435 (62.0)
Ever	5262 (28.1)	1493 (38.0)
Past	2411 (13.0)	651 (16.6)
Current	2805 (14.9)	838 (21.3)
Heavy/Risky Drinking	3446 (19.2)	847 (21.7)
BMI, kg/m^2		
<18.5 (score = 1)	1002 (5.1)	128 (3.2)
18.5-24.9 (score=1)	9314 (49.6)	2050 (51.9)
25-<30 (score = 0.5)	5057 (27.3)	1098 (27.8)
≥30 (score = 0)	3199 (18.0)	672 (17.0)
Physical activity, MET-h/wk		
0 (score = 0)	6480 (33.8)	1235 (31.3)
3-6 (score = 0.5)	4661 (24.9)	1018 (25.8)
9-12 (score = 1)	4522 (24.7)	1012 (25.6)
15-21 (score = 1)	2949 (16.5)	684 (17.3)

BMI = Body mass index. GED=General educational development. IQR=interquartile range. MET=metabolic equivalent of task, hours per week.

Supplementary Table 9. Statistical analysis of differences in absolute excess risk for chronic health conditions with moderately unhealthy or unhealthy lifestyle score between childhood cancer survivors and sibling controls compared using bootstraps.

	Lifestyle score	Survivors	Siblings	P
**Chronic health conditions**		**AER**	**AER**	
Hypertension	Moderate Unhealthy	27.1	13.5	0.014
	Unhealthy	34.6	43.9	0.036
Dyslipidemia	Moderate Unhealthy	11.4	8.6	0.21
	Unhealthy	18.4	26.1	0.017
Diabetes mellitus	Moderate Unhealthy	15.0	9.0	0.017
	Unhealthy	26.2	22.2	0.24
Heart attack	Moderate Unhealthy	2.3	4.7	0.029
	Unhealthy	6.3	5.6	0.69
Heart failure	Moderate Unhealthy	3.8	-0.9	0.020
	Unhealthy	5.4	2.0	0.021
Stiff or leaky valves	Moderate Unhealthy	4.1	-1.4	<0.001
	Unhealthy	4.4	-1.5	<0.001
Arrhythmia	Moderate Unhealthy	4.0	0.7	0.016
	Unhealthy	4.1	3.3	0.57
Stroke	Moderate Unhealthy	2.5	1.0	0.14
	Unhealthy	0.9	3.0	0.014
Joint replacement	Moderate Unhealthy	1.7	3.4	0.082
	Unhealthy	3.8	4.6	0.52
Osteoporosis	Moderate Unhealthy	3.1	1.4	0.12
	Unhealthy	0.9	3.4	0.006
Respiratory disease	Moderate Unhealthy	1.8	2.4	0.65
	Unhealthy	3.8	4.0	0.90
Subsequent malignant neoplasms	Moderate Unhealthy	6.4	-1.7	0.080
	Unhealthy	-2.8	2.7	0.14
**Emotional distress and quality of life**				
Anxiety	Moderate Unhealthy	1.1	1.1	0.94
	Unhealthy	2.8	1.4	<0.001
Depression	Moderate Unhealthy	1.4	1.1	<0.001
	Unhealthy	3.4	1.8	<0.001
Mental component	Moderate Unhealthy	3.1	2.5	<0.001
	Unhealthy	8.5	5.6	<0.001
Physical component	Moderate Unhealthy	4.0	3.1	<0.001
	Unhealthy	8.9	7.1	<0.001

All statistical tests were two-sided. No correction was performed for multiple testing since all outcomes were distinct hypotheses of individual interest. Nonparametric bootstrap was used to test statistical significance of the differences. Number of participants in the total population is 18,664, with analysis of each outcome differing slightly due to pre-existing conditions, as reported in Supplementary Table 1.
AER = Absolute excess risk

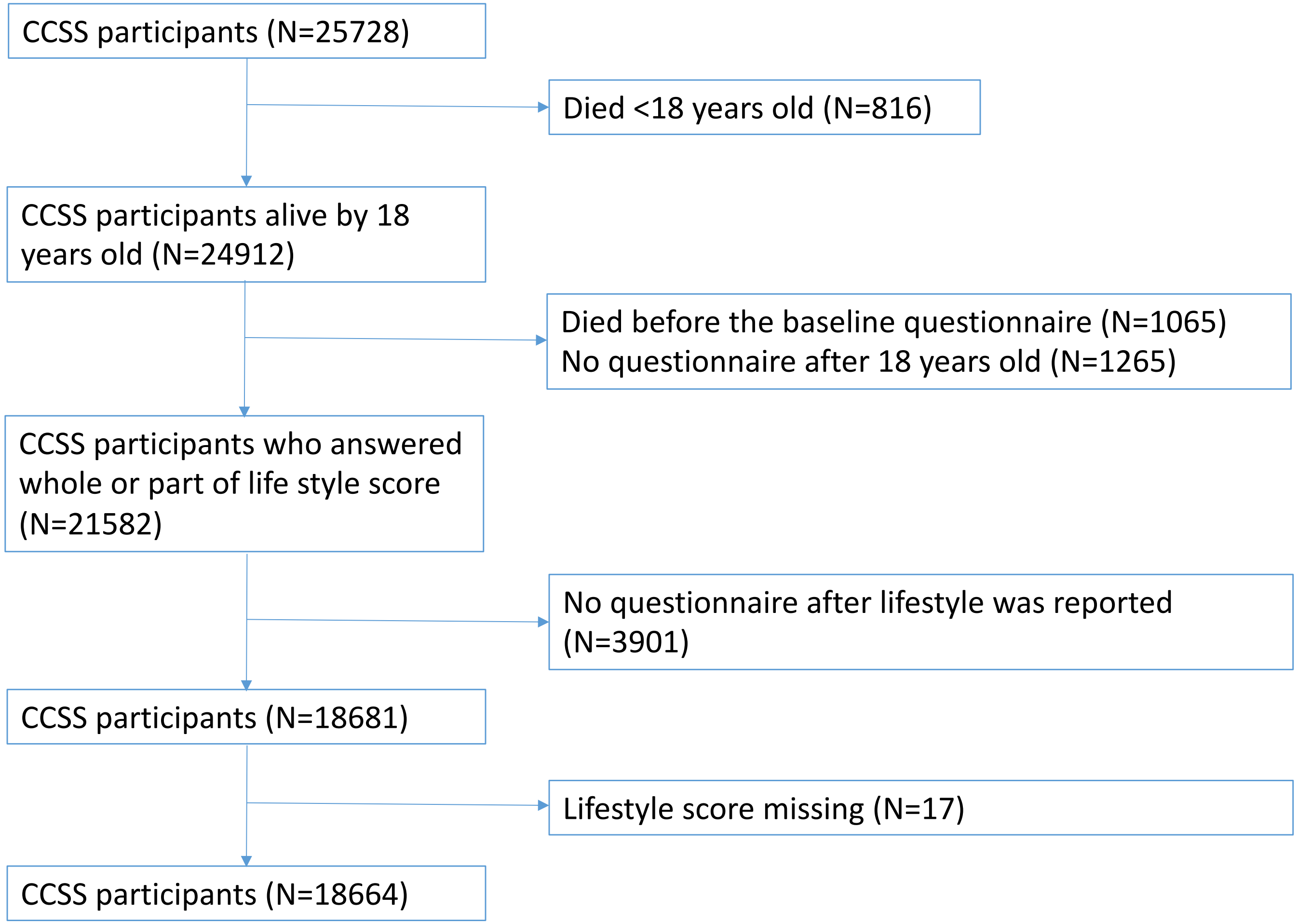

Supplementary figure 1. Flow chart of the childhood cancer survivor study population.

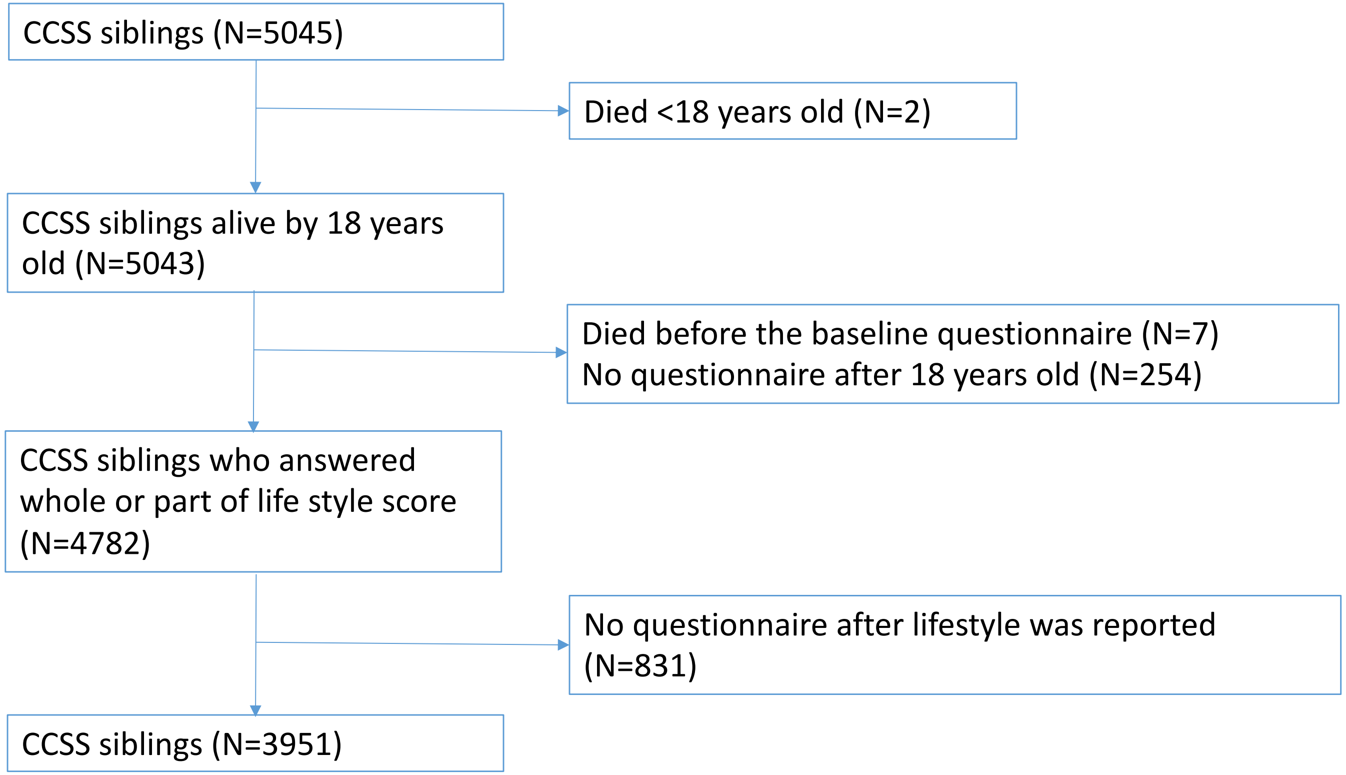

Supplementary figure 2. Flow chart of the sibling comparators study population.
